# Using opinion leaders to address intervention gaps in concussion prevention in youth sports: key concepts and foundational theory

**DOI:** 10.1186/s40621-018-0158-7

**Published:** 2018-07-09

**Authors:** Zachary Y. Kerr, Johna K. Register-Mihalik, Juliet Haarbauer-Krupa, Emily Kroshus, Vivian Go, Paula Gildner, K. Hunter Byrd, Stephen W. Marshall

**Affiliations:** 1 Department of Exercise and Sport Science, University of North Carolina, 313 Woollen Gym CB#8700, Chapel Hill, NC 27599-8700 USA; 2 Injury Prevention Research Center, University of North Carolina, CVS Plaza, Suite 500, 137 East Franklin Street, CB#7505, Chapel Hill, NC 27599-7505 USA; 3 Department of Exercise and Sport Science, University of North Carolina, 125 Fetzer Hall CB#8700, Chapel Hill, NC 27599-8700 USA; 4 Division of Unintentional Injury, National Center for Injury Prevention and Control, Centers for Disease Control and Prevention, 4700 Buford Highway, MS F-62, Atlanta, GA 30341 USA; 5 Department of Pediatrics, University of Washington, 2001 Eighth Ave, Seattle, WA 98121 USA; 6 Seattle Children’s Research Institute; Child Health, Behavior and Development, 2001 Eighth Ave, Suite 400, Seattle, WA 98121 USA; 7 Department of Health Behavior, Gillings School of Global Public Health, University of North Carolina, 361 Rosenau Hall CB#7440, Chapel Hill, NC 27599-7440 USA; 8 Department of Epidemiology, Gillings School of Global Public Health, University of North Carolina, CVS Plaza, Suite 500, 137 East Franklin Street, CB#7505, Chapel Hill, NC 27599-7505 USA

**Keywords:** Injury prevention, Socio-ecological model, Pro-equity intervention, Public health

## Abstract

Behavioral interventions to increase disclosure and proper management of concussion in youth sports have unrealized potential when it comes to preventing concussion. Interventions have focused on changing individual athlete behavior and have fallen short of the potential for sustained systemic behavioral change. One potentially critical reason for this shortfall is that other key determinants of risk behaviors at all levels of the socio-ecological model (e.g. interpersonal, community, policy) are not addressed in extant programming. There is a critical need for theory-driven interventions that address concussion prevention and education at the community level and target sustainable culture change. The Popular Opinion Leader (POL) intervention, a multi-level intervention model previously successfully employed in multiple public health contexts, is theoretically well positioned to affect such change. POL is based on the Diffusion of Innovations framework and involves identifying, recruiting, and training well-respected and trusted individuals to personally endorse prevention and risk-reduction within their social networks. Critical behavioral changes related to concussion disclosure and management have been shown to diffuse to others if enough opinion leaders endorse and support the behaviors. This article summarizes the concepts and principles of POL and describes how it could be adapted for and implemented in youth sport settings. For optimal impact, POL needs to adapt to several factors unique to youth sports settings and culture. First, adult involvement may be important, given their direct involvement in the athlete’s medical care. However, parents and coaches’ opinions on injury care-seeking, competition, and safety may affect their perceptions of POL. Second, youth sports are structured settings both physically and socioculturally. Games and practices may provide opportunities for the informal interactions that are critical to the success of POL. However, youth sport setting membership is transient as players get older and move to other sport settings; POL approaches need to be self-sustaining despite this turnover. Moreover, stakeholder value placed on athlete development and competition, alongside safety, must be considered. Formative research is needed to ensure that POL principles are translated into the youth sport setting while maintaining fidelity to the concepts and principles that have made POL successful for other health outcomes.

## Background

Concussion has been documented in emergency department (ED) populations (Bakhos et al. 2010; Coronado et al. 2015; Bryan et al. 2016) and youth (Bryan et al. 2016; Dompier et al. 2015), high school (Marar et al. 2012; O’Connor et al. 2017), collegiate (Zuckerman et al. 2015), and professional (Benson et al. 2011; Green et al. 2015; Orchard et al. 2013; Clark et al. 2017) sport settings. Public concern has been intensified by recent research suggesting potential short- and long-term effects associated with recurrent concussion and head impact exposure in current and former athletes (Guskiewicz et al. 2003, 2005, 2007; McCrea et al. 2003).

Despite this increased public awareness, prevention efforts are hindered by notable gaps in our knowledge around: (Bakhos et al. 2010) head impact prevention strategies and (Coronado et al. 2015) injury identification, in the context of youth populations. Of note, identification of the injury remains a challenge as previous research has identified ranges of 35-62% of concussions among high school, collegiate, and professional athletes going unreported and therefore unmanaged (Kerr et al. 2014). Although research has focused on increasing concussion knowledge and awareness at the youth sport level (Register-Mihalik et al. 2017; Kroshus et al. 2015a), and policy/legislation has been introduced at the state and organization levels to mitigate concussion risk (Pop Warner Football 2018; Little League 2016), increased knowledge and awareness does not necessarily translate to better reporting behaviors. Further, interventions that use a community-level approach to concussion identification and prevention are limited. Such interventions would likely benefit from considering the tenets of the socio-ecological model, which posits that there are determinants of behavior at multiple levels distal to the individual (e.g., interpersonal, environment, legislative) (Stokols 1992).

This article proposes the adaption of the Popular Opinion Leader (POL) intervention to the problem of concussion in youth sports. Based on the Diffusion of Innovations framework (Rogers 2010), the POL model involves training groups of influential individuals to have conversations in which they personally endorse key prevention and management messages within their social networks. When enough opinion leaders endorse and support the desired behaviors, behavioral changes have been shown to diffuse to others – potentially leading to changes in community norms (Kelly et al. 1991; Coker et al. 2017; Lomas et al. 1991; Wiist and Snider 1991). We begin by summarizing selected key aspects of the state of youth sports concussion prevention. We then introduce and describe the theoretical basis of the POL intervention, and provide areas of context that should be considered for implementing such a POL program in the diverse and challenging setting of youth sports. For this paper, we define youth as <13 years of age.

## Key Issues in youth sports concussion prevention relevant to behavioral interventions

### Magnitude and diversity of the problem of youth sports concussion

Youth sports concussion has a large incidence, with estimates suggesting that 1.1 to 1.9 million sport-related traumatic brain injuries are sustained each year by US children (Bryan et al. 2016). However, there remains a substantial gap in the literature with respect to determining the incidence of concussion in youth sports overall, particularly in sports other than football. Concussions occur in a variety of settings, including high schools, middle schools, club sports, recreational leagues, and informal sports activities. However, only in the past five years have quality data on youth level concussion estimates been collected and published (Table 1). Examining concussion risk relative to other settings is made more difficult by the lack of studies as well as the potential differences in methodologies among studies that may bias comparisons (Kerr et al. 2017a).

**Table 1:** Estimated concussion incidence from studies reporting youth data, 2011 and after

Authors	Timeframe	Sport	Age Range (years)	Concussion Rates
Kontos et al. (2013)	2011	Football	8-12	1.76/1000AE
Dompier et al. (2015)	2012-2013	Football	5-15	0.99/1000AE
Kerr et al. (2016a)	2012-2014	Football	5-15	0.87/1000AE
Kerr et al. (2015a)	2014	Football	5-15	0.62/1000AE
Kontos et al. (2016)	2013/14-2014/15	Ice Hockey	12-18	1.58/1000AE
Kerr et al. (2017b)	2015	Boys’ Lacrosse	9-15	0.84/1000AE
O’Kane et al. (2014)	2008-2012	Girls’ Soccer	11-14	1.2/1000 hours
Beachy and Rauh (2014)	1998-2008	29 Boys’ and Girls’ Sports	12-15	Sport-specific rates reported with football being the highest (0.35/1000AE)
Kerr et al. (2017c)	2015/16	12 Boys’ and Girls’ Sports	11-13	Overall rate of 0.75/1000AE, with highest rates reported in football (2.61/1000AE) and girls’ soccer (1.30/1000AE)

### Intervention strategies that integrate primary, secondary, and tertiary prevention

From a public health perspective, although primary prevention of concussion in youth sports aims to reduce the incidence of concussion, it is also very important to consider the secondary and tertiary intervention strategies that include a range of targets for reducing the risk and consequences of concussion injury (Table 2). For such a complex problem as concussion, intervention strategies likely need to be applied at many points across the natural history of concussion with the goal of preventing and mitigating progression into adverse outcomes for youth involved in sports. This framework also ensures that those who were not protected by primary prevention means have additional opportunities to ensure proper detection and management of their concussions. Thus, alongside reducing concussion risk, education and behavioral modifications should also improve the on-site and subsequent management of concussion so that the likelihood of long-term adverse effects is reduced.

**Table 2:** Public health model of concussion prevention

Stage	Strategies	Example
Primary	Strategies to prevent injury occurrence	Eliminate or limit contact in sports gameplay and training
Secondary	Manage injury to prevent worsening of condition	On-site management of concussion
Tertiary	Prevent long term complications and reoccurrence of injury	Medical recommendation for delayed return to sport or disqualification due to sustaining multiple concussions

Table is not exhaustive

### Too much emphasis on the individual and policy, not enough emphasis on community relationships and dynamics

As concussion-focused interventions develop primary, secondary, and tertiary prevention strategies, there is also a need to focus on those levels of influence within the socio-ecological model that have yet to be addressed, particularly the interpersonal relationships among youth sports stakeholders and the social environment (e.g., norms) that exist within this setting (Kerr et al. 2014). Research has also focused on increasing the knowledge and awareness of concussion signs, symptoms, and management strategies, among athletes, administrators, coaches, and parents in youth sports (Kerr et al. 2014; Register-Mihalik et al. 2017; Kroshus et al. 2015a). Meanwhile, policy/legislation has been introduced at the state and organization levels to mitigate concussion risk (Pop Warner Football 2018; Little League 2016). While such research on the individual levels of intervention has been highly beneficial and informative alongside the passage of policy/legislation, there has been little or no emphasis on interventions that consider the interpersonal relationships among all the youth sport stakeholders (e.g., among youth sport athlete parents, between parent and athlete) and apply a community-level approach to prevention and injury identification. However, we lack research or established intervention paradigms on how to comprehensively shift sport cultures when it comes to concussion safety.

### Athlete and community equity

The culture and resources of a given youth sport setting play a critical role in concussion prevention and identification. As such, there are equity concerns about the implementation of concussion interventions. Social inequalities in concussion prevention related to lower socio-economic status have been documented (Lin et al. 2015; Kroshus et al. 2017a), potentially due to less access to resources, lower levels of general health literacy, and additional fees and extra costs that may inhibit participation. Large-scale implementation of effective interventions could potentially exacerbate current health inequalities if adoption or implementation is unequal across communities (Frohlich and Potvin 2008; Bernard et al. 2007; Lorenc et al. 2013). Intervention programs should utilize formative research to better understand factors that drive inequities and to provide guidance on promoting adoption and implementation in diverse settings. Although school sports tend to provide a setting that better represents the general population, school-funding mechanisms tied to community property taxes help produce disparities in school resources (Kroshus et al. 2017b). Consequently, as seen in the high school setting (Kroshus et al. 2017b), youth sport settings in more affluent communities may have higher quality facilities and equipment and are also more likely to employ athletic trainers, who play a key role in concussion education, recognition, and management. To limit variable implementation by community resources, it is necessary to ensure that the intervention was scalable at relatively modest cost and could be supported by what available resources were readily available.

## Rationale for considering a popular opinion leader framework

Given the magnitude and complexities of concussion in youth sport, a concussion intervention needs to be applicable to the context of youth sports concussions; address both primary prevention (e.g., head impact reduction) and secondary prevention (e.g., management of concussion); be capable of effecting change at the community, environmental, and legislative levels; and respond to social equity concerns (Table 3). A public health intervention model used in other domains (Kelly et al. 1991) that shows promise in terms of meeting these criteria is the Popular Opinion Leader (POL) intervention. Based on the Diffusion of Innovations framework (Rogers 2010), this model involves groups of influential individuals, defined as those who are trusted and respected by others, in spreading key messaging with a goal of changing the norms in their respective communities. Key behavioral changes in a population can be initiated and has been shown to diffuse to others if enough opinion leaders within the population are known to adopt, endorse, and support the behavior. Opinion leader interventions have been widely used in other areas of public health (Kelly et al. 1991; Coker et al. 2017; Lomas et al. 1991; Wiist and Snider 1991) but to date have not been applied in youth sport settings.

**Table 3:** Characteristics of an intervention that aims to reduce the incidence and severity of sports concussion in a youth sports setting

- Be applicable to the national context of youth sports concussion, which has an estimated incidence of over a million injuries per year (Bryan et al. 2016) in a wide variety of settings, many of which do not have onsite health care providers	
- Be capable of effecting change at multiple levels of the socio-ecological model (individual, interpersonal, community/environmental, and legislative)	
- Address both concussion risk reduction such as head impact reduction (primary prevention) and management of concussion (secondary prevention), since narrowly focused programs will not be adopted and maintained by sports communities and will not fully address concussions from the prevention and care standpoint	
- Be flexible enough to adapt to diverse settings, so that social equity concerns are not exacerbated by the intervention	

**Fig. 1:**
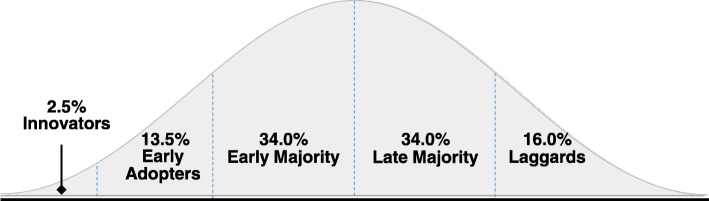
Diffusion of Innovation framework (adapted from Rogers 2010). The framework centers on factors that affect adoption of ideas within the entire community. Engaging innovators and early adopters, those of whom that have early “buy-in” of the idea, will help to accelerate the dissemination process. The use of opinion leaders, who are perceived as being able to exert a large influence on the attitudes of individuals, may equate to a greater likelihood of buy-in to the idea and consequently behavior change

Opinion leader interventions tend to be based on the Diffusion of Innovation framework (Rogers 2010), (Fig. 1) focusing on factors that affect adoption of ideas within an entire community (i.e., change in cultural norms). Individuals within a setting are divided into five groups: innovators, early adopters, early majority, late majority, and laggards, all of whom adopt the idea at different stages. Engaging innovators or “opinion leaders,” community members with early “buy-in” of the idea has been shown to accelerate dissemination and the time at which saturation of the idea occurs (Valente and Pumpuang 2007). Opinion leader interventions, such as POL, involve groups of trusted and respected individuals who are recruited and trained to conduct public health outreach by changing the norms in their communities. An opinion leader intervention is scalable and can be deployed at relatively low-cost in a large number of settings. Because adoption of ideas occurs in phases (Rogers 2010), continued recruitment of opinion leaders for buy-in of the idea helps cultivate changes in cultural norms on an ongoing basis and can help with sustainability.

Adaption and implementation of the opinion leader approach to the problem of youth sports concussion requires development of a carefully planned intervention strategy, followed by testing of the strategy and refinement of the approach. Fortunately, some of the complexities of the intervention have been refined in other public health settings and these lessons can be applied to youth sport, while recognizing that population-specific adaptations will likely need to be made. The opinion leader strategy has been utilized to decrease high-risk sexual practices (Kelly et al. 1991), sexual violence (Coker et al. 2017), caesarian births (Lomas et al. 1991), and adolescent smoking (Wiist and Snider 1991).

**Fig. 2:**
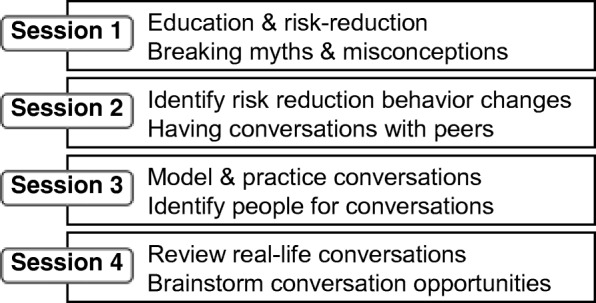
Sessions included in Popular Opinion Leader (POL) intervention (content adapted from Kelly et al. 1991). The sessions provide opinion leaders with education related to the outcome of interest and provide training on how opinion leaders can have casual conversations with peers in their social networks about their personal endorsements of risk reduction related to the outcome of interest

The most widely used POL intervention focuses on HIV risk reduction in men-who-have-sex-with-men (MSM) (Kelly et al. 1991). In one study, trusted and influential individuals in the Lesbian, Gay, Bisexual, and Transgender (LGBT) community in Biloxi, MS, were first identified as opinion leaders, and then recruited, and trained to have casual conversations with peers in their social networks about their personal endorsements of HIV risk reduction (Fig. 2) (Kelly et al. 1991). The opinion leaders were identified through ethnographic assessments, coupled with interviews with “gatekeepers” or individuals within the settings with an apt knowledge of who were the trusted and influential individuals. In the original study, the “gatekeepers” were the bartending staff that were employed within the bar in which the study was focused (Kelly et al. 1991).

In the three-to-six-month follow-up, community-level reductions in unprotected anal intercourse and increases in condom use were observed (Kelly et al. 1991). The POL intervention has been successfully used in numerous MSM communities (NIMH Collaborative HIV/STD Prevention Trial Group 2010; Somerville et al. 2006; Kelly et al. 1997) and international settings (NIMH Collaborative HIV/STD Prevention Trial Group 2010), and among low SES women (Sikkema et al. 2000), Latino migrant workers (Somerville et al. 2006), alcohol users (Sivaram et al. 2004), and injection drug users (Latkin 1998). Currently, the CDC includes the POL intervention as part of its efforts to reduce HIV infections (Centers for Disease Control and Prevention 2015). Furthermore, to better aid proper implementation, follow-up research has identified nine core elements of dissemination of the POL intervention (Table 4) (Kelly 2004).

**Table 4:** Core elements of the Popular Opinion Leader (POL) intervention (adapted from Kelly et al. 1991)

1	Direct the intervention to an identifiable target population in well-defined community setting where population’s size can be estimated
2	Use ethnographic techniques systematically to identify those persons who are most popular, influential, and trusted by others (i.e., conduct community identification)
3	Over life of program, train 15% of the target population as opinion leaders
4	Teach opinion leaders skills for initiating risk-reduction messages to peers during everyday conversations
5	Teach opinion leaders characteristics of effective behavior change communication targeting risk-reduction attitudes, norms, intentions, and self-efficacy; have opinion leaders endorse, in conversations, the benefits of safer behavior and recommend practical steps needed to implement change
6	Hold weekly meetings of groups of opinion leaders in sessions that use instruction, facilitator modeling, and extensive role-playing exercises to help opinion leaders refine their skills and gain confidence in delivering effective HIV prevention messages to others
7	Have opinion leaders set goals to engage in risk-reduction conversations with friends and acquaintances in the target population between weekly sessions
8	Review, discuss, and reinforce outcomes of opinion leaders’ conversations at subsequent training sessions
9	Use logos, symbols, or other devices as conversation starters between the opinion leaders and others

## Adapting the POL intervention to the youth sport setting

Much of the theory and strategy underlying the work of the original POL intervention can be readily adapted to the youth sport setting simply by shifting the focus from HIV transmission to concussion prevention. Table 5 provides the preliminary framework for the adaption of POL to youth sports concussion. Whereas the HIV-specific POL interventions have recruited the majority of opinion leaders from the at-risk population (i.e. MSM) (Kelly et al. 1991), a POL intervention adapted to the youth sport setting could theoretically benefit from also recruiting administrators, coaches and parents alongside youth athlete opinion leaders. At this developmental stage for youth, adults still play a critical role in influencing youth opinions (Lau et al. 1990; Bauer et al. 2011; Lake et al. 2004; Kelly et al. 2002).

**Table 5:** Strengths of Popular Opinion Leader (POL) Intervention and applicability to youth sport setting

Strength	As applied to youth sport setting
Community-level reductions in HIV-related risk behaviors (Kelly et al. 1991)	May lead to a preventive sport culture that mitigates negative norms and beliefs that may increase concussion risk in athletes
Used in settings of varying socio-economic statuses, race/ethnicity, and urbanicity (Kelly et al. 1991; NIMH Collaborative HIV/STD Prevention Trial Group 2010; Somerville et al. 2006; Kelly et al. 1997; Sikkema et al. 2000; Latkin 1998)	Formative research is an essential aspect of POL intervention to ensure that it can be an effective means by which sport safety culture changes can occur in multiple youth sport settings
Provides education and dispels myths and misconceptions (Kelly 2004)	Intervention includes: education about the incidence, diagnosis, management, and prevention of concussion; and the promotion of safer game play
Concurrently considers individual, interpersonal, and environmental levels of influence (Kelly et al. 1991)	Training athletes, coaches, and parents to disseminate knowledge across multiple youth sport stakeholders and change cultural norms within all stakeholder-specific networks
Personal endorsements from influential community members regarding risk reduction behaviors (Kelly et al. 1991)	Can advance community-level knowledge of primary, secondary, and tertiary concussion prevention strategies, while correcting related myths and misconceptions
Dissemination framework of nine core elements (Kelly 2004)	Formative research and fidelity measures assess compliance with core elements and identify implementation factors specific to youth sports and concussion
Relies on community to disseminate and maintain cultural norm changes (Kelly 2004)	Intervention follows core elements to ensure a sufficient number of appropriate opinion leaders are identified and recruited

To implement the POL in the youth sport setting, groups of trusted and influential individuals, including athletes, parents, and coaches, need to be identified, recruited, and trained to have casual, one-on-one conversations with friends, peers, and teammates in their social networks. “Gatekeepers” used to identify potential opinion leaders are likely to vary by setting but may include administrators, teachers, and on-site medical staff. Potential opinion leaders could also be identified by asking teams who they would trust to provide information and asking members of the community if they would be willing to share information related to concussion prevention and sports safety.

The opinion leader training sessions would follow the template used in the original study in which sessions first provided education related to concussion and strategies of good communication (Fig. 2) (Kelly et al. 1991). The conversations that opinion leaders would have within their social networks would focus on the endorsement of strategies for safer practice and competition play, including disclosing suspected concussion symptoms and seeking proper management for suspected concussions. During these conversations, opinion leaders would correct misperceptions and discuss the importance of safer practice and competition play, and proper management of concussions. They would 1) communicate their personal approval of such tenets of player safety, and 2) discuss the improvement in performance associated with improved playing techniques, as even young athletes often value performance above safety (Register-Mihalik et al. 2013).

A comprehensive understanding of the community in which an intervention will occur is central to the POL intervention model (Kelly et al. 1991; Kelly 2004). Given that the youth sport setting differs in several important respects from the previous LGBT/MSM communities that have successfully incorporated the POL model, it is important to consider how such factors may alter and affect implementation in comparison to previous research. Ethnographic research, inclusive of interviews and organizational scans, are integral to ensuring that the POL intervention will approach concussion prevention in a manner that is relevant to the stakeholders in a particular youth sport setting. Given these concerns, we recommend formative research to examine how the adaption of a POL intervention in the youth sport setting can best adhere to the basic tenets of the original intervention while considering the unique aspects of youth sports.

There is a multitude of issues associated with adapting an intervention from one setting to another. However, we believe that a starting point of focus should examine: coach and parental involvement; the structured and hierarchal nature of the youth sports setting that inherently places emphasis on athlete development; and the transient nature of youth sports as players get older and move to other sport settings. These topics are further discussed in detail below.

### Integrating adults as opinion leaders in the youth sport setting

**Fig. 3:**
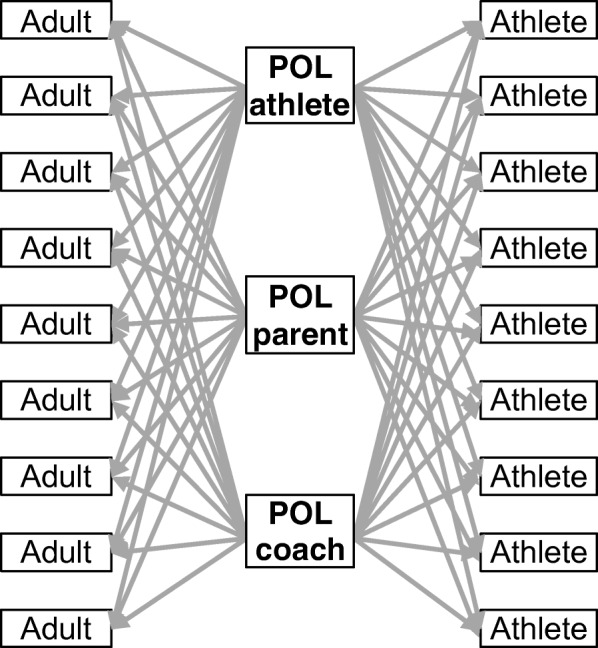
Initial dissemination of information from popular opinion leaders (POL) to youth sport setting

We believe an adaption of the POL intervention to youth sports will likely benefit from selecting opinion leaders from a larger pool of stakeholders than would be the case in the MSM communities. This includes adult stakeholders such as coaches and parents. Alongside the youth athletes and coaches, they form what is known as the athletic triangle (Blom et al. 2013). Although the goal of the POL intervention is to impact youth athlete behavior, doing so requires shaping the knowledge, attitudes, and behaviors of everyone within a given youth sport culture, including coaches and parents. In addition to their role in communicating with athletes, using both parents and coaches as opinion leaders strengthens the dissemination of concussion prevention and care-seeking messages across and within the different youth sports stakeholder groups. (Fig. 3). Approaching different youth sports stakeholder groups may also aid in still ensuring dissemination of the POL intervention if one group is not as invested (e.g., parents invested, but not coaches). With this, the POL intervention would still operate with consideration of the socio-ecological model given that athletes (i.e., the individual level) and their interpersonal communication with their parents. Nevertheless, “buy-in” from numerous stakeholders may aid in dissemination of the intervention. As a result, process evaluation is warranted to identify factors that facilitate and impede “buy-in”.

#### Coach roles in the sport setting

Coaches serve as a fundamental source of influence of youth athletes, particularly in creating a team culture in which care-seeking and treatment adherence are perceived as positive outcomes (Barnett et al. 1992; Poczwardowski et al. 2002; Koester 2000; Nixon and Howard 1994; Podlog and Dionigi 2010). Current research has focused on two lines of research concerning coaches and concussion-related safety outcomes. These areas suggest that coaches, as key players in an intervention program, may have positive effects on concussion safety outcomes of interest. The first evaluates interventions that use coaches to help disseminate concussion education and prevention. Sarmiento et al. (Sarmiento et al. 2010) noted that high school coaches liked the content of the CDC HEADS UP toolkit. Yet, Sawyer et al. (Sawyer et al. 2010) reported that only 7.2% of coaches had actually disseminated toolkit information to other school staff, athletes, and parents (Sawyer et al. 2010). Researchers have also evaluated USA Football’s “Heads Up Football” (HUF) coaching education program. Although youth football leagues adopting HUF had fewer head impacts in practice (Kerr et al. 2015b), concussions rates did not differ from “non-HUF” leagues. One study using a small sample of Indiana high school football teams with a USA Football-trained player safety coach had lower concussion rates in practices than teams with coaching education only (Kerr et al. 2016b).

The second line of research has focused on coaches’ roles in encouraging and not stigmatizing disclosure and care-seeking for suspected concussions (Kroshus et al. 2015a, b; Baugh et al. 2014; Kroshus et al. 2015c; Kroshus et al. 2017c). At the collegiate level, even though medical staff are primarily involved in concussion education, athletes reported wanting their coach to be involved in sharing information about concussion (Kroshus and Baugh 2016). Athletes that perceived support from their coaches were also more likely to report concussion symptoms (Baugh et al. 2014). A recent study with collegiate wrestling coaches supported the use of autonomy-supportive behaviors such as providing choice, avoiding controlling behaviors, and acknowledging athlete feelings to help encourage communication of concussion safety from coach to athlete (Kroshus et al. 2017c). Although such research has largely focused on the high school and collegiate levels and needs to be expanded to look at those at the youth level, the findings illustrate that approaches beyond instilling concussion-related knowledge are necessary to support concussion safety.

#### Parental roles in the sport setting

Parents are viewed as important in influencing not only youth athlete motivation but also achievement in sports (Blom et al. 2013; Harwood and Swain 2001; Lavoi and Stellino 2008) and sportsmanship (Shields et al. 2007). However, unlike coaches, few studies or interventions have targeted parents. As part of their child’s involvement with sports, parents can also develop social networks with other parents on the team and be influenced by these interactions (Dorsch et al. 2009). Although most of the research on socialization has been conducted on younger children (Morrongiello and House 2004; Morrongiello and Schwebel 2008; Peterson et al. 1995; Peterson and Stern 1997), an understanding of this process builds the basis for conceptualizing the parent-child interaction for injury response in middle school children. At the same time, there is limited research on parent-focused intervention programs in youth sports. Thus, concussion prevention interventions should consider the role of parents to target issues such as youth attitudes, behaviors, intentions to disclose a concussion, and to address knowledge, perceived controls, and normative perceptions in youth athletes regarding concussion.

Parents are also the primary socialization agent for injury risk and response in children. Parents’ decisions to seek medical assessment after sports concussion may involve several factors, including their past experiences and approaches to sports (Becker and Maiman 1975; Committee on Sports Medicine and Fitness 2000), their attitudes about their child’s sport (Kroshus et al. 2017d), their child’s past healthcare use (Janicke et al. 2001) demographic factors (i.e. marital status, race and ethnicity, access and insurance coverage) (Zandieh et al. 2009), and other less-explored factors such as their own healthcare experiences. Parent income and educational levels also contribute to their understanding of injury risk (Lin et al. 2015; Bloodgood et al. 2013).

### Considering the physical and sociocultural structure of the youth sport setting

Youth sports, particularly those in school settings, have structured aspects within their organization. This includes physical structures associated with the youth sport setting (e.g., game and practice schedules), as well as sociocultural structures related to all facets that may be considered integral to youth sports, including athlete development and competition. A successful adaption of the POL intervention needs to be cognizant of the general and site-specific structures of the youth sport setting, yet also develop strategies to maximize facilitators and minimize barriers to proper implementation.

#### Using games and practices as opportunities to have conversations for opinion leaders

Youth sports are structured around scheduled training and competition events. Such a schedule of sport-based events may provide infrastructure to have the informal interactions desired within the POL intervention. These opportunities should also exist in recreational youth leagues, where the focus may be more on skills development and practices than competitions. Formative research should include a focus on identifying times, places, and contexts in which youth sport stakeholders can have conversations with one another about concussion. One possibility is that youth athletes need transportation from guardians to and from practice most days, which may not be present among older adolescent athletes who are able to drive. Parents that stay or come to these practices may also have opportunities to engage in conversation with other parents. Also, if carpooling is used to aid transportation needs, parents could also have conversations with athletes besides their own children.

#### Acknowledging adults’ motivations for and investments in their children’s sport participation

It is important to consider how differing motivations for sport participation intersect with willingness to engage with the POL intervention or general discussions about safety. The focus of some parents and coaches may be more focused on athlete development and competition, more so than social networking and interaction. Prior research suggests that parents who more strongly value their child’s sport achievement are less likely than other parents to talk about concussion safety with their child (Kroshus et al. 2017d)**.** Although potential opinion leaders may be influential and trusted, they may not be ready to advocate all aspects of concussion prevention or risk reduction. Thus, as potential opinion leaders are recruited and introduced to the intervention during the first session, it is important to provide baseline concussion and sport safety education and to gauge their interest and concerns regarding continuing through the subsequent sessions. Further, in reality, opinion leaders that are skeptical at first, but then become more invested in the intervention as it progresses, may actually be beneficial as they will consequently be able to discuss their own progression and resulting buy-in in their conversations within their social networks (Maiorana et al. 2007).

The competitive nature of sports may also impact the acceptance of the intervention. This may occur as athletes compete for a starting position or when parents push for their children to have more playing time than others. It is possible that communication about health and safety with opinion leaders may be perceived to have ulterior motives. For example, parents may misinterpret an opinion leader’s discussion of health and safety as an attempt to have a child removed from play to increase playing time for their own child. To address this issue, the messaging that is used by opinion leaders should not focus on specific concussions sustained by children from other families, but rather personal experiences with concussion prevention strategies that can reflect how the opinion leaders support safer gameplay and concussion management. In addition, the POL intervention sessions that allow opinion leaders to practice having personally endorsed conversations should also provide time for them to brainstorm how to approach the many unique issues within this setting.

#### Changing and maintaining cultural norms in a transient population

A community is a dynamic population in which individuals will come and leave. The MSM communities used in the original POL studies (Kelly et al. 1991, 1997) were likely more stable than youth sport settings. Membership in youth sports settings is necessarily transient as players will get older and be required to move to other sport settings. Likewise, parents move from these same sport settings with their children. Also, there tends to be a high rate of youth sport coach turnover (Smith and Smoll 2011; Woods 2015). Consequently, a POL intervention needs to be self-sustaining despite this turnover between seasons, and more importantly, capitalize on this opportunity for additional dissemination as opinion leaders transition to new sports settings. This may involve identifying more stable positions (e.g., school administrator) that while not necessarily appropriate to be opinion leaders themselves, may be able to function as a champion for the program across time. To ensure local sustainability as stakeholders within youth sports change, transfer of institutional knowledge should be situated within the existing infrastructure of the specific youth sport setting (e.g., team manager leads the process).

Consistent with the socio-ecological model, opinion leaders may also more readily adopt and maintain a program that is supported by the school or sports league. School-level support could include ensuring that school employees are engaged in the program. For example, school-based certified medical professionals such as athletic trainers may themselves be well placed to serve as opinion leaders and to assist in identifying, recruiting, and training other opinion leaders. Not all schools have certified athletic trainers, and because of different school sizes, resources, and organizational structures there is a critical need to develop flexible school-level implementation strategies that are adaptable to the wide range of economic and social factors that are characteristic of our education systems.

## Conclusions

The strong and well-replicated evidence base from the HIV literature suggest POL interventions are a promising means of shifting the norms of a sports community so that community members increasingly adopt concussion prevention- and sport safety-related policies and practices. The youth sports community comprises well-integrated groups with a strong sense of identity and structure. The athletes involved are minors that require coach supervision and often have parents present at the majority of events. A POL intervention could utilize key social referents including athletes, parents, and coaches to converse with individuals at all levels of the youth sport programs in an effort to diffuse key information that encourages safer play and improved post-concussion recognition and response. Formative research is needed to ensure that the intervention is adapted to consider barriers relevant to each unique youth sport setting, while ensuring preservation of key POL principles that underpin the success of this intervention.
